# Prognostic value of summed motion score assessed by gated SPECT myocardial perfusion imaging in patients with dilated cardiomyopathy

**DOI:** 10.3389/fcvm.2023.1144333

**Published:** 2023-03-15

**Authors:** Jun-Yan Zhu, Xin-Chao Wang, Nan Huang, Xiao-Qian Li, Yan Cheng, Zhi-Fang Wu, Yuan-Yuan Li, Ping Wu, Li Li, Hua Wei, Si-Jin Li, Ji-Min Cao

**Affiliations:** ^1^ Key Laboratory of Cellular Physiology at Shanxi Medical University, Ministry of Education, Taiyuan, China; ^2^ Department of Physiology, Shanxi Medical University, Taiyuan, China; ^3^ Department of Nuclear Medicine, First Hospital of Shanxi Medical University, Taiyuan, China; ^4^ School of Public Health, Shanxi Medical University, Taiyuan, China; ^5^ Collaborative Innovation Center for Molecular Imaging of Precision Medicine, Shanxi Medical University, Taiyuan, China; ^6^ Department of Critical Care Medicine, First Hospital of Shanxi Medical University, Taiyuan, China

**Keywords:** dilated cardiomyopathy, prognostics, outcome, wall motion, gated SPECT

## Abstract

**Background:**

The prognosis of patients with dilated cardiomyopathy (DCM) is poor and new indicators are urgently needed to predict lethal cardiac events. This study aimed to investigate the value of summed motion score (SMS) in predicting cardiac death of DCM patients using gated single photon emission computed tomography (SPECT) myocardial perfusion imaging (MPI).

**Methods and results:**

Eighty-one patients with DCM who underwent ^99m^Tc-MIBI gated SPECT MPI were retrospectively enrolled and were divided into cardiac death and survivor groups. The functional parameters of left ventricle including SMS were measured using quantitative gated SPECT software. During the follow-up period of 44 (25, 54) months, 14 (17.28%) cardiac deaths were observed. Compared with the survivor group, SMS was significantly higher in the cardiac death group. Multivariate cox regression analysis showed that SMS was an independent predictor for cardiac death (HR 1.34, 95% CI 1.02–1.77, *P* = 0.034). SMS also provided incremental prognostic value over other variables in the multivariate model as determined by likelihood ratio global chi-squared test. In the Kaplan-Meier survival analysis, the event-free survival rate was significantly lower in the high-SMS (HSMS) group than the low-SMS (LSMS) (log-rank *P *< 0.001). Furthermore, the area under curve (AUC) of SMS was larger than that of LVEF at the 12th month of follow-up (0.85 vs. 0.80, *P* = 0.045).

**Conclusion:**

SMS is an independent predictor of cardiac death in DCM patients and provides incremental prognostic value. SMS might have higher predictive value than LVEF for early cardiac death.

## 1. Introduction

Dilated cardiomyopathy (DCM) is one of the most common causes of heart failure, second only to ischemic cardiomyopathy, and is the most common indication for heart transplantation worldwide (1, 2). DCM has a poor prognosis, with a 1-year mortality rate of 25%–30% and a 5-year survival rate of 50%. Despite advances in DCM treatment, the 10-year survival rate is less than 60% (2, 3). Current studies have shown that many variables are associated with adverse DCM outcomes, and the functional parameters of left ventricle obtained by whatever imaging techniques remain the major prognosticators, but their values in estimating the prognosis of DCM are limited (2, 4). Therefore, there is still an urgent need for new markers to predict the outcome of DCM patients so as to prolong their survival.

Studies have shown that regional wall motion obtained from gated single photon emission computed tomography (SPECT) myocardial perfusion imaging (MPI) provides important prognostic information on cardiovascular outcome events (5–7). For example, in patients with known or suspected coronary artery disease (CAD), abnormalities of post-stress reversible regional wall motion are the most powerful predictive parameters of cardiac events including cardiac death, nonfatal myocardial infarction, unstable angina, and early or late coronary revascularization (8). Furthermore, SPECT has the advantage of high reproducibility and repeatability and can be used for semi-quantitative analysis of ventricular wall motion to obtain summed motion score (SMS) (9–11). SMS can be considered as an independent prognostic factor in the risk stratification of patients with suspected CAD (12). SMS can also be used as a predictor of cardiac events and have a value for clinical risk stratification of diabetic patients with normal perfusion (13). However, to date, there is no report documenting the value of SMS in predicting the prognosis of DCM. Accordingly, this study aimed to investigate the value and incremental value of SMS in the prognosis of patients with DCM using gated SPECT MPI.

## 2. Materials and methods

### 2.1. Patient population

Eighty-one patients with DCM were retrospectively enrolled in this study at the First Hospital of Shanxi Medical University from January 2015 to July 2020. These patients were eligible to be enrolled after their diagnosis were confirmed, based on medical history, clinical, electrocardiographic, and echocardiographic findings according to the recommendation criteria (14–16). Patients with the following conditions were excluded (17, 18): (1) CAD with lumen stenosis greater than 50% or a history of myocardial infarction or stent placement; (2) valvular heart disease, alcoholism, inflammatory cardiomyopathy, or specific cardiomyopathy secondary to any known systemic disease; (3) patients with a history of cardiac resynchronization therapy or implantable cardioverter defibrillator; (4) patients with malignant tumors. All the enrolled patients underwent coronary angiography or coronary computed tomography angiography, with coronary artery stenosis ≤50%, and had complete data of demography, electrocardiography, echocardiography, and gated SPECT MPI. The study was approved by the Ethics Review Committee of the First Hospital of Shanxi Medical University, and informed consent was obtained from all patients.

### 2.2. Acquisition of gated SPECT MPI image

The ^99m^Tc was provided by Beijing Atomic High-tech Co. LTD, with radiochemical purity >95%; MIBI was purchased from Jiangsu Institute of Atomic Medicine. To perform gated SPECT MPI, each patient underwent intravenous injection of 20–30 mCi of ^99m^Tc-MIBI in an overnight fast state, followed by a fatty meal 20 min after tracer injection, and resting gated SPECT MPI scan was performed 60 min after injection. The MPI images were acquired on a dual-collimator instrument (Siemens Symbia T16, Siemens) using a standard protocol. Image acquisition parameters: SMART-ZOOM collimator, energy peak 140 keV, matrix 128 × 128, magnification 1.0, acquisition of myocardial images in ECG gated tomography mode, and ECG window width 20%. The two probes were rotated at an angle of 76° and images were acquired total for 208° from a right anterior oblique 38° to a left posterior oblique 66°. The acquisition speed was 25 sec per frame, and 34 frames were acquired in approximately 8 min. The ordered-subsets expectation maximization (OSEM) method was used to reconstruct the image (iteration number 12, subset 5) and to obtain the myocardial tomographic images at the left ventricular (LV) short axis, horizontal long axis and vertical long axis.

### 2.3. Quantitative analysis of gated SPECT MPI image

Gated SPECT tomograms were reconstructed and reoriented using automated software (Autoquant software, Cedars Sinai Medical Center, Los Angeles, California, United States). A 17-segment model (American Heart Association, AHA) was used to analyze the gated SPECT data (19, 20). Quantitative gated SPECT (QGS) software was used to analyze: (1) LV global functional parameters, including LV ejection fraction (LVEF) (LVEF was also measured by conventional echocardiography in this study), LV end diastolic volume (EDV), and LV end systolic volume (ESV); (2) LV regional function parameters, including regional myocardial wall motion (RWM) and regional myocardial wall thickening (RWT); (3) LV mechanical systolic synchrony parameters: phase standard deviation (PSD), phase histogram bandwidth (PBW), mean and phase entropy (PE). Segmental wall motion was graded according to the 6-point scoring system (0-normal, 1-mild hypokinesia, 2-moderate hypokinesia, 3-severe hypokinesia, 4-akinesia, 5-dyskinesia). Wall thickening was graded using the 4-point scoring system (0-normal, 1-mild, 2-moderate to severe, 3-absent) (12, 21). The scores of the 17 segments were added up to obtain the summed motion score (SMS) and summed thickening score (STS), respectively (13).

### 2.4. Follow-up after gated SPECT MPI

Follow-up data were obtained through phone contact with patients or their relatives, and other information of patients were obtained from the records of the hospitalized case system. The endpoint was cardiac death, including cardiac arrest or death from circulatory failure occurring within the first hour or refractory chronic heart failure (17). The mean follow-up period was 44 (25, 54) months.

### 2.5. Statistical analysis

R4.1.2 software and IBM SPSS Statistics 28.0 were used for statistical analysis. Package “survminer” was used to find the cut-off value for SMS, and the population was divided into high-SMS (HSMS) and low-SMS (LSMS) groups based on the cut-off value of SMS. The quantitative data adopted the Mann–Whitney *U* test and were expressed as M (P25, P75). The categorical variable adopted the chi-square test or nonparametric Fisher’s exact test and was presented as number and percentage.

The Kaplan-Meier method was used to obtain the event-free survival curve, and the log-rank test was used for comparison; univariable cox survival analysis and least absolute shrinkage and selection operator (LASSO) regression were performed to assess the association of each variable with survival outcome. The method of LASSO was used to select LV function parameters. Univariable cox regression analysis was used to calculate the hazard ratio (HR) and the 95% confidence interval (95% CI) of each variable. Multivariable cox regression analysis was used to determine independent predictors of adverse events. The deviation residuals were drawn for continuous variables to check the hypothesis of proportional hazards. Variables with *P* < 0.05 in univariable cox regression analysis were included in multivariable Cox regression analysis. For any variable included in the cox model, the proportional hazard assumption was not rejected. In addition, Spearman’s correlation was used to illustrate the strength of an association between imaging indicators.

We also used global likelihood-ratio Chi-square statistics to evaluate the incremental value of different models including clinical variables (clin) only, clin + SMS, clin + conventional LV function parameters (con LV), and clin + SMS + con LV. In addition, time-dependent receiver operating characteristic (ROC) curves were used to evaluate the performance of the indicators; Package “time ROC” was used to draw ROC curves between different imaging indicators in different times and to compare the area under the curve (AUC) of them. *P* < 0.05 was considered to be statistically significant.

## 3. Results

### 3.1. Patient characteristics

Table 1 presents the demographic and clinical characteristics of the 81 participating patients [54.0 (42.0; 62.0) years old, 72.8% male]. Patients were divided into two groups: the cardiac death group and the survivor group. Patients in the cardiac death group were older and had lower BMI than those in the survivor group (*P* < 0.05). Of all the patients, 4 (5.0%) were classified as having NYHA functional class I, 10 (12.3%) as NYHA class II, 39 (48.1%) as NYHA class III, and 28 (34.6%) as NYHA class IV. All patients received guideline-directed medication for heart failure. The use of *β*-blocker in the survivor group was significantly higher than that in the cardiac death group (47, 70.1% vs. 5, 35.7%, *P* = 0.033). There was no significant difference in the use of ACE inhibitors/ARBs, diuretics and digoxin between the two groups. There was also no significant difference in hypertension, diabetes, hyperlipidemia, LBBB, mitral regurgitation and LVEF measured by echocardiography (LVEF_echo_) between the two groups. The QRS duration was longer in the cardiac death group than in the survivor group (130.0 [106.0; 165.0] ms vs. 100.0 [90.5; 113.0] ms, *P* = 0.007).

**Table 1:** Baseline characteristics of the survivors and the cardiac death cases.

Variables	All (*N = 81*)	Survivors (*N = 67*)	Cardiac deaths (*N = 14*)	*P* value
Age (years)	54.0 [42.0; 62.0]	51.0 [41.5; 61.0]	65.0 [54.0; 70.0]	0.003
Male (*n*, %)	59 (72.8%)	48 (71.6%)	11 (78.6%)	0.748
BMI (kg/m^2^)	25.0 [22.2; 27.5]	25.4 [22.8; 28.9]	22.2 [21.2; 25.6]	0.008
Hypertension	25 (30.9%)	21 (31.3%)	4 (28.6%)	1.000
Diabetes	19 (23.5%)	15 (22.4%)	4 (28.6%)	0.730
Hyperlipidemia	18 (22.2%)	15 (22.4%)	3 (21.4%)	1.000
QRS duration (ms)	102.0 [92.0; 121.5]	100.0 [90.5; 113.0]	130.0 [106.0; 165.0]	0.007
LBBB	11 (13.6%)	8 (11.9%)	3 (21.4%)	0.346
NYHA class I/II/III/IV	4/10/39/28	3/8/33/23	1/2/6/5	0.865
Medication				
ACE inhibitors/ARBs	60 (74.1%)	50 (74.6%)	10 (71.4%)	0.750
*β*-blockers	52 (64.2%)	47 (70.1%)	5 (35.7%)	0.033
Diuretics	65 (80.2%)	55 (82.1%)	10 (71.4%)	0.460
Digoxin	38 (46.9%)	32 (47.8%)	6 (42.9%)	0.968
Echocardiography				
Mitral Regurgitation	43 (53.1%)	34 (50.7%)	9 (64.3%)	0.529
LVEF (%)	28.0 [23.0; 32.0]	29.0 [24.0; 33.5]	26.0 [23.0; 31.0]	0.263

Data are expressed as medians (interquartile ranges) or number (percentage). BMI, body mass index; LBBB, left bundle branch block; NYHA, New York Heart Association; ACE, angiotensin-converting enzyme; ARB, angiotensin II receptor blocker; LVEF, left ventricular ejection fraction.

**Figure 1:**
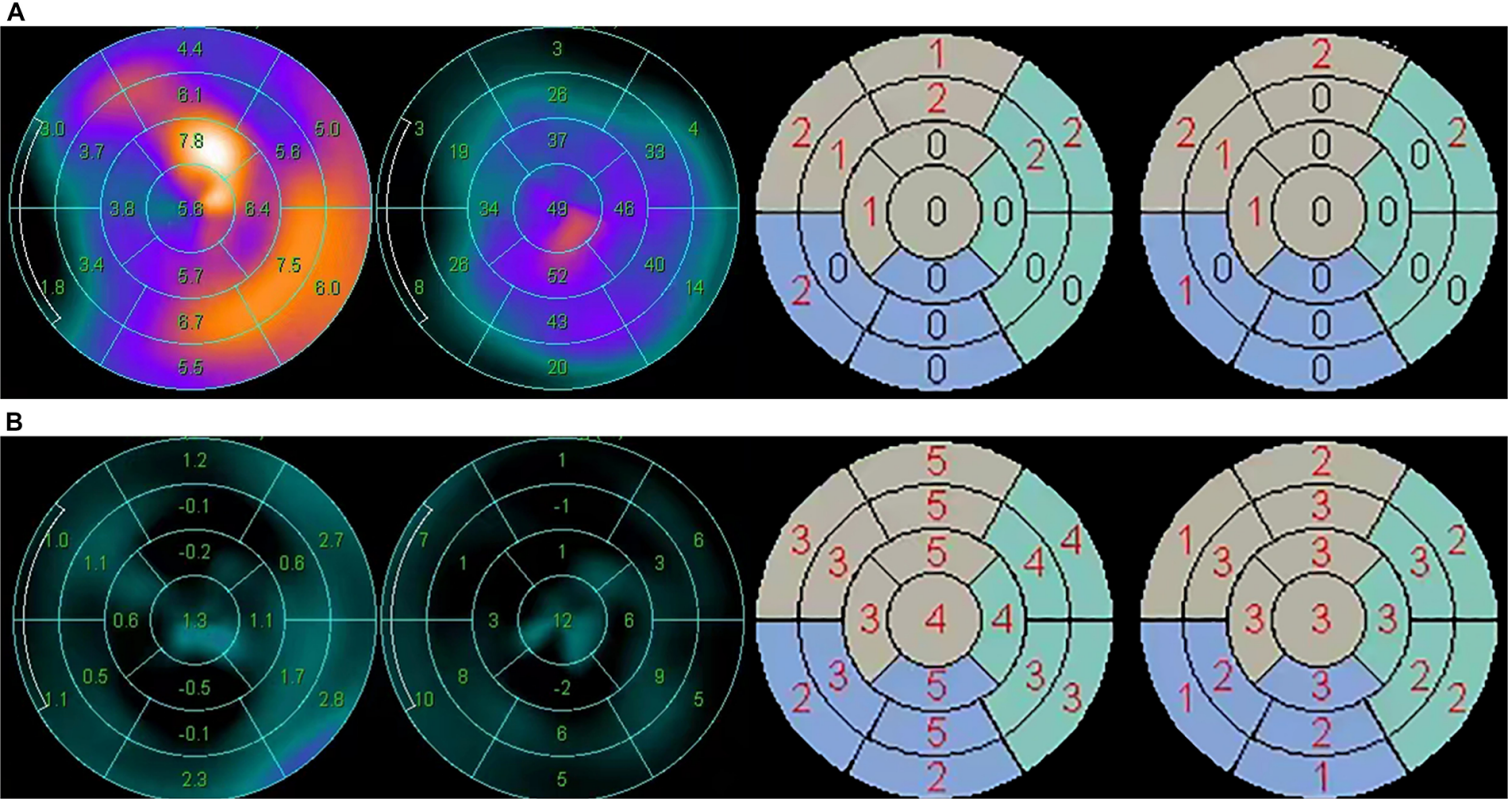
Wall motion and wall thickening analysis in two typical cases of the survivor group and the cardiac death group, respectively. (**A**) The wall motion (mm), wall thickening (%), wall motion score and wall thickening score of a 72-year-old male patient in the survivor group. SMS = 13, STS = 9. (**B)** The wall motion (mm), wall thickening (%), wall motion score and wall thickening score of a 65-year-old male patient in the cardiac death group. SMS = 63, STS = 39.

Table 2 describes the LV function parameters from gated SPECT MPI. Compared with the survivor group, the cardiac death group had significantly lower LVEF (13.0 [9.00; 15.5]% vs. 18.0 [15.0; 25.0]%, *P* = 0.001) and greater EDV and ESV (EDV: 298.0 [254.0; 385.0] ml vs. 197.0 [166.0; 260.0] ml, *P* = 0.001; ESV: 258.0 [224.0; 348.0] ml vs. 159.0 [125.0; 220.0] ml, *P* = 0.001). Compared with the survivor group, the cardiac death group had higher SMS (56.5 [53.0; 58.8] vs. 47.0 [37.5; 53.5], *P* < 0.001), higher STS (34.0 [32.2; 37.0] vs. 31.0 [27.5; 33.5], *P* = 0.003), and larger PE (66.0 [63.5; 72.5] vs. 62.0 [55.0; 67.0], *P* = 0.015). There was no significant difference in other mechanical contraction synchrony parameters PBW, PSD and mean between the two groups. Figure 1 shows the wall motion and wall thickening of a representative case in both groups.

**Table 2:** Gated SPECT MPI LV function parameters.

Variables	All (*N* = 81)	Survivors (*N* = 67)	Cardiac deaths (*N* = 14)	*P* value
EDV (ml)	218.0 [174.0; 276.5]	197.0 [166.0; 260.0]	298.0 [254.0; 385.0]	0.001
ESV (ml)	175.0 [143.5; 236.0]	159.0 [125.0; 220.0]	258.0 [224.0; 348.0]	0.001
LVEF (%)	17.0 [12.5; 22.5]	18.0 [15.0; 25.0]	13.0 [9.00; 15.5]	0.001
SMS	50.0 [40.0; 55.0]	47.0 [37.5; 53.5]	56.5 [53.0; 58.8]	<0.001
STS	32.0 [28.0; 34.0]	31.0 [27.5; 33.5]	34.0 [32.2; 37.0]	0.003
PBW (°)	96.0 [66.0; 132.0]	96.0 [66.0; 132.0]	108 [81.0; 141.0]	0.296
Mean	146.5 [138.3; 161.7]	146.4 [137.4; 157.4]	155.0 [143.9; 164.3]	0.103
PSD (°)	27.0 [19.8; 34.7]	26.7 [19.0; 34.1]	28.0 [22.3; 36.8]	0.385
PE	63.0 [56.0; 68.0]	62.0 [55.0; 67.0]	66.0 [63.5; 72.5]	0.015

Data are expressed as medians (interquartile ranges). MPI, myocardial perfusion imaging; LV, left ventricle; STS, summed thickening score; EDV, end diastolic volume; ESV, end systolic volume; PBW, phase histogram bandwidth; PSD, phase standard deviation; PE, phase entropy.

Table 3 shows the baseline characteristics and LV function parameters of patients grouped according to SMS. Patients were dichotomized into two groups according to the cut-off value of SMS: 44 patients were assigned to the LSMS group (≤50), and 37 patients were assigned to the HSMS group (>50). Compared with the LSMS group, patients in the HSMS group had lower BMI (*P* = 0.035), longer QRS duration (*P* = 0.019), more NYHA class III/IV cases (*P* = 0.045) and lower LVEF_echo_ (*P* = 0.001). There was no statistical difference in age, sex, hypertension, diabetes, hyperlipidemia, LBBB, mitral regurgitation and medication use (ACE inhibitors/ARBs, *β*-blockers, diuretics and digoxin) between LSMS and HSMS groups. Compared with the LSMS group, the HSMS group had significantly lower LVEF (*P* < 0.05), significantly higher SMS (*P* < 0.001), and significantly larger values of EDV, ESV, STS, mean and PE (*P* < 0.05). The differences in PBW and PSD between the LSMS and HSMS groups were not statistically significant.

**Table 3:** Baseline characteristics and LV function parameters in LSMS and HSMS groups.

Variables	All (*n* = 81)	LSMS group (*n* = 44)	HSMS group (*n* = 37)	*P* value
Age (years)	54.0 [42.0; 62.0]	54.5 [43.8; 62.5]	54.0 [40.0; 61.0]	0.345
Male (*n*, %)	59 (72.8%)	29 (65.9%)	30 (81.2%)	0.201
BMI (kg/m^2^)	25.0 [22.2; 27.5]	25.5 [22.9; 28.9]	24.2 [22.2; 26.2]	0.035
Hypertension	25 (30.9%)	13 (29.5%)	12 (32.4%)	0.969
Diabetes	19 (23.5%)	12 (27.3%)	7 (18.9%)	0.535
Hyperlipidemia	18 (22.2%)	12 (27.3%)	6 (16.2%)	0.355
QRS duration (ms)	102.0 [92.0; 121.5]	98.0 [89.0; 111.0]	110.0[96.0; 137.0]	0.019
LBBB	11 (13.6%)	3 (6.8%)	8 (21.6%)	0.053
NYHA class I/II/III/IV	4/10/39/28	3/9/20/12	1/1/19/16	0.045
Medication				
ACE inhibitors/ARBs	60 (74.1%)	29 (65.9%)	31 (83.8%)	0.115
*β*-blockers	52 (64.2%)	28 (63.6%)	24 (64.9%)	1.000
Diuretics	65 (80.2%)	35 (79.5%)	30 (81.1%)	1.000
Digoxin	38 (46.9%)	18 (40.9%)	20 (54.1%)	0.338
Echocardiography				
Mitral Regurgitation	43 (53.1%)	23 (52.3%)	20 (54.1%)	1.000
LVEF (%)	28.0 [23.0; 32.0]	30.0 [26.8; 36.0]	25.0 [23.0; 29.0]	0.001
SPECT MPI LV function parameters				
EDV (ml)	218.0 [174.0; 276.5]	192.0 [160.0; 236.0]	266.0 [199.0; 320.0]	<0.001
ESV (ml)	175.0 [143.5; 236.0]	148.0 [116.0; 194.0]	233.0 [169.0; 269.0]	<0.001
LVEF (%)	17.0 [12.5; 22.5]	21.5 [18.0; 27.0]	12.0 [9.0; 15.0]	<0.001
SMS	50.0 [40.0; 55.0]	40.5 [33.8; 47.0]	55.0 [53.0; 59.0]	<0.001
STS	32.0 [28.0; 34.0]	28.0 [24.8; 31.2]	35.0 [33.0; 37.0]	<0.001
PBW (°)	96.0 [66.0; 132.0]	93.0 [64.5; 134.0]	108 [90.0; 138.0]	0.139
Mean	146.5 [138.3; 161.7]	142.0 [136.0; 152.0]	158.0 [144.0; 166.0]	0.001
PSD (°)	27.0 [19.8; 34.7]	26.8 [17.4; 32.7]	30.0 [23.9; 36.9]	0.050
PE	63.0 [56.0; 68.0]	57.0 [53.0; 64.4]	67.0 [63.0; 72.0]	<0.001

Data are expressed as medians (interquartile ranges) or numbers (percentage). BMI, body mass index; LBBB, left bundle branch block; NYHA, New York Heart Association; ACE, angiotensin-converting enzyme; ARB, angiotensin II receptor blocker; LVEF, left ventricular ejection fraction; MPI, myocardial perfusion imaging; LV, left ventricle; LSMS, low-summed motion score; HSMS, high-summed motion score; STS, summed thickening score; EDV, end diastolic volume; ESV, end systolic volume; PBW, phase histogram bandwidth; PSD, phase standard deviation; PE, phase entropy.

### 3.2. Prediction of cardiac death

**Figure 2:**
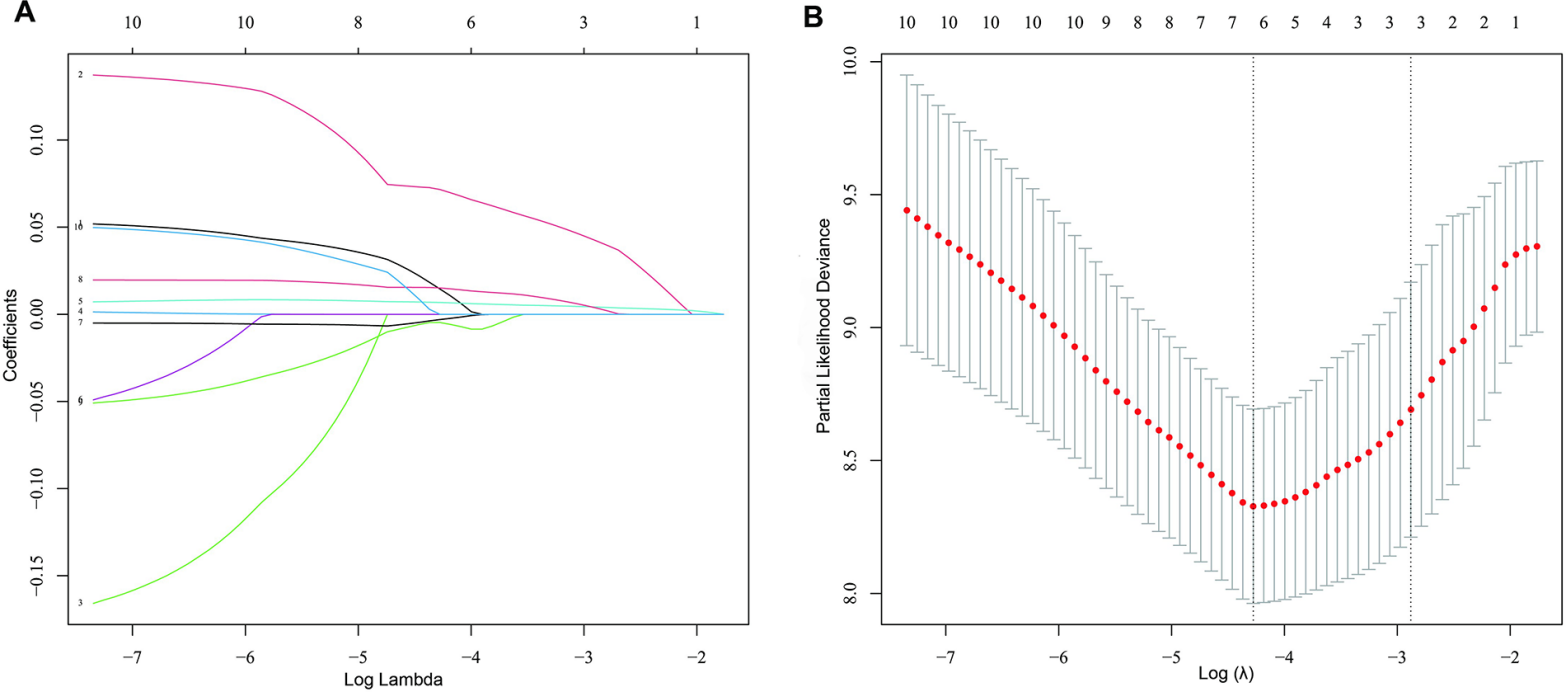
Screening for variables using the LASSO regression model. (**A)** Results of the Lasso regression. The 10 colored lines marked by Arabic numerals represent 10 different variables, lines 1–10 represented LVEF_echo_, SMS, STS, EDV (ml), ESV (ml), LVEF (%), PBW (°), Mean, PSD (°), and PE, respectively. By the LASSO regression, three variables (SMS, ESV and mean) were finally selected from the 10 variables. (**B)** LASSO coefficient profiles of the 10 prognostic factors for cardiac death. The optimal value of *λ* was determined by 10-fold cross-validation to find the minimum mean squared error (minMSE) + 1 standard error of minMSE backwards along the *λ* path.

Univariable cox regression analysis showed that age, BMI, QRS duration (ms), *β*-blockers, EDV, ESV, LVEF, SMS, STS and PE were associated with cardiac death (total *P* < 0.05) (Table 4). These parameters were included in the multivariate analysis. In addition, LASSO regression could select variables by performing a penalized regression on all variable coefficients so that coefficients of relatively insignificant independent variables became zero. To eliminate possible collinearity among LV function parameters, we used LASSO regression to screen variables (Figure 2), and finally selected SMS, ESV and mean from 10 variables and then put these three variables into the multivariate cox regression model. The multivariate cox regression analysis revealed that SMS was an independent predictor of cardiac death (HR 1.34, 95% CI 1.02–1.77, *P* = 0.034). Age and *β*-blockers were also influential factors for cardiac death (Age: HR 1.08, 95% CI 1.01–1.15, *P* = 0.017; *β*-blockers: HR 0.13, 95% CI 0.02–0.76, *P* = 0.024) (Table 4).

**Table 4:** Cox regression analysis for cardiac death.

Variable	Univariate analysis		Multivariate analysis	
	HR (95% CI)	*P* value	HR (95% CI)	*P* value
Age (years)	1.05 (1.01–1.09)	0.016	1.08 (1.01–1.15)	0.017
BMI (kg/m^2^)	0.78 (0.63–0.95)	0.015		
QRS duration (ms)	1.02 (1.01–1.04)	0.011		
*β*-blockers	3.22 (1.08–9.63)	0.037	0.13 (0.02–0.76)	0.024
EDV (ml)	1.01 (1.00–1.01)	0.001		
ESV (ml)	1.01 (1.00–1.01)	0.000		
LVEF (%)	0.82 (0.72–0.93)	0.002		
SMS	1.12 (1.05–1.20)	0.001	1.34 (1.02–1.77)	0.034
STS	1.20 (1.25–1.37)	0.008		
PE	1.09 (1.02–1.17)	0.013		
Mean	1.03 (0.99–1.06)	0.119		

The variables with *P *< 0.05 from univariate analysis or variables with non-zero coefficients from Lasso regression were put into the multivariable Cox regression model. HR, hazard ratios; CI, confidence interval.

**Figure 3:**
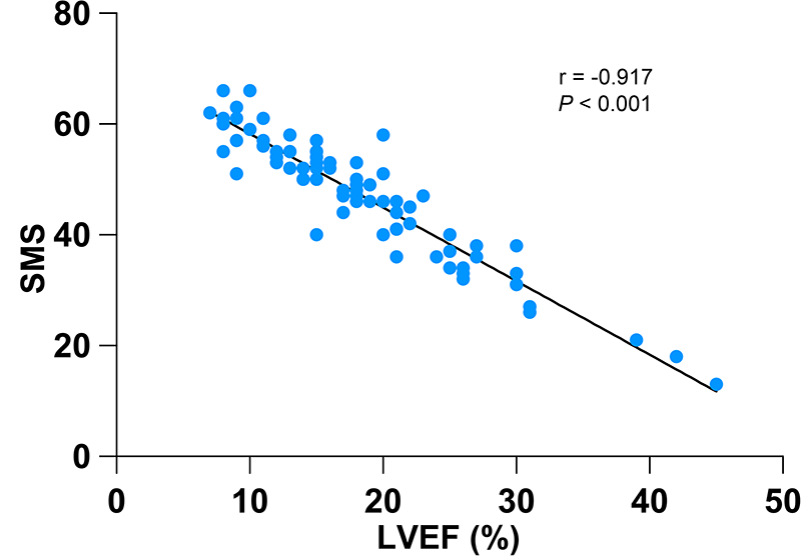
Relationship between SMS and LVEF. A significant inverse correlation was observed between SMS and LVEF (*r* = −0.917, *P* < 0.001).

Table 5 shows the hazard ratio (HR) of SMS on cardiac death after correction for potential confounders including age, gender, BMI, hypertension, diabetes, hyperlipidemia, QRS duration, NYHA class, ACE inhibitors/ARBs, *β*-blockers, diuretics, digoxin, LVEF_echo_, EDV, ESV, LVEF, STS, PBW, mean, PSD and PE. In addition, our results showed that SMS was significantly associated with LVEF (*r* = −0.917, *P* < 0.001) (Figure 3). To clarify whether the effect of SMS on the prognosis was influenced by LVEF, we further explored the interaction between SMS and LVEF, but did not find interaction between them in model 3 (*P* = 0.972) (Table 5), indicating that SMS was always an independent predictor of cardiac death in DCM patients (total *P* < 0.05).

**Table 5:** Association of cardiac death with SMS.

Model	HR (95% CI)	*P* value	*P* value for interactor^#^
1	1.191 (1.085,1.322)	<0.001	
2	1.357 (1.143,1.613)	0.001	
3	3.267 (1.256,8.497)	0.015	0.972

Model 1 was adjusted with age, gender, BMI, Hypertension, Diabetes, Hyperlipidemia, QRS duration and NYHA class; Model 2 was adjusted with ACE inhibitors/ARBs, *β*-blockers, Diuretics and Digoxin which were added to model 1; Model 3 was adjusted with echocardiographic LVEF and SPECT MPI LV function parameters, including EDV, ESV, LVEF, STS, PBW, Mean, PSD and PE which were added to model 2.

^#^ The *P* value for the interactor of SMS × LVEF.

### 3.3. Incremental prognostic value of SMS for cardiac death

**Figure 4:**
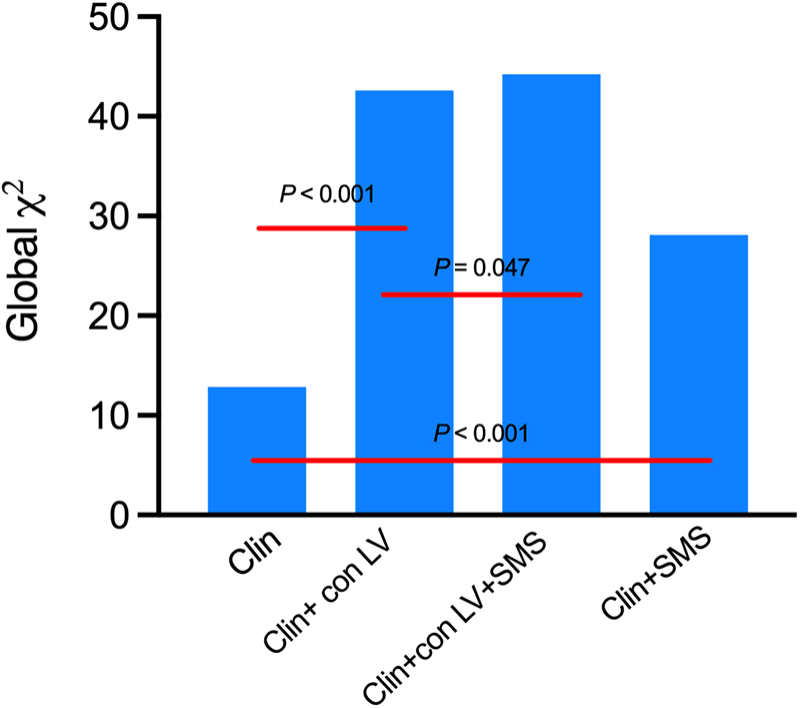
Incremental association between clinical variables (Clin), conventional LV function parameters (con LV), SMS and cardiac death as indicated by the statistics in serial multivariable adjusted models. Clinical variables include age, gender, BMI, Hypertension, Diabetes, Hyperlipidemia, QRS duration and NYHA class. LV index included EDV, ESV, LVEF, STS, Mean and PE.

Adding SMS increased the global Chi-square from 12.845 to 28.120 (*P* < 0.001) and adding the conventional LV function parameters (con LV) increased the global Chi-square from 12.845 to 42.624 (*P* < 0.001) compared to clinical variables only. Adding SMS to the model that included clinical variables and con LV still improved the global Chi-square (42.624 vs. 44.240, *P* = 0.047) (Figure 4).

### 3.4. Survival analysis

**Figure 5:**
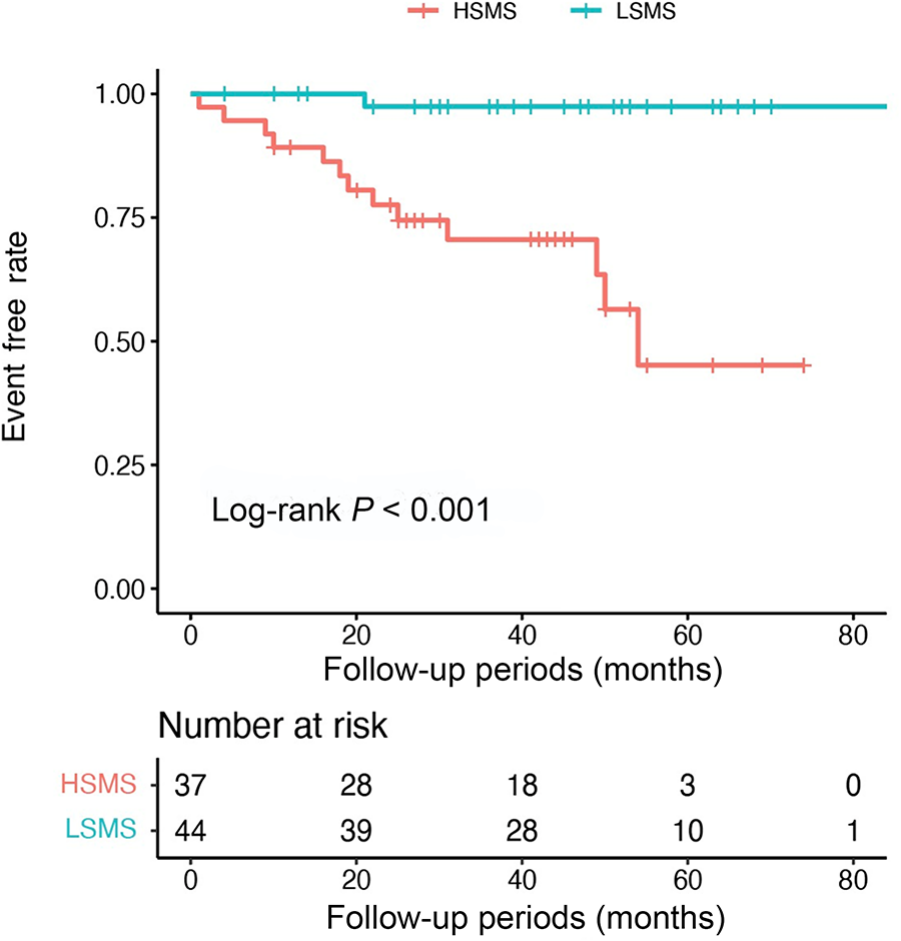
The Kaplan-Meier event-free survival curve.

**Figure 6:**
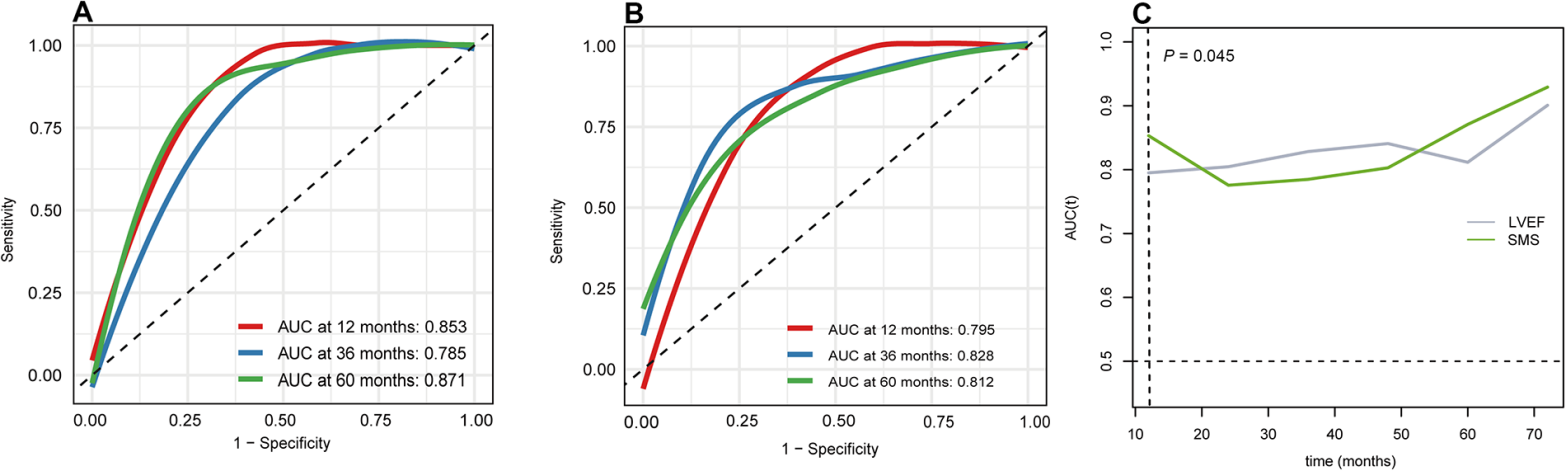
Time-independent receiver operating characteristic (ROC) curve. (**A)** ROC curve of SMS in different time. (**B)** ROC curve of LVEF in different time. (**C)** the AUC of SMS and LVEF in different time.

During the follow-up period of 44 (25, 54) months, cardiac death occurred in 14 (17.28%) of the total 81 patients. The Kaplan-Meier event-free survival curve showed that the HSMS group had a lower event-free survival rate than the LSMS group (log-rank *P* < 0.001). The higher the SMS, the worse the prognosis (Figure 5). The time-dependent ROC curve of SMS showed that the area under the curve (AUC) at 12 months, 36 months and 60 months was 0.853, 0.785 and 0.871, respectively (Figure 6A). The time-dependent ROC curve of LVEF revealed that the AUC at 12 months, 36 months and 60 months was 0.795, 0.828 and 0.812, respectively (Figure 6B). The time-dependent ROC curves showed that the AUC of SMS was larger than that of LVEF at the 12th month of follow-up (*P* = 0.045), and there was no significant difference in the AUC between SMS and LVEF at other follow-up times (Figure 6C).

## 4. Discussion

In this study, we investigated the prognostic value of SMS obtained by gated SPECT MPI in patients with DCM. To our knowledge, we demonstrated for the first time that SMS is an independent predictor of cardiac death in patients with DCM. On the basis of clinical variables and conventional LV functional parameters, SMS may also provide incremental value in predicting cardiac death in patients with DCM. In addition, we demonstrated that SMS may be more predictive of early cardiac death compared with LVEF.

Many previous studies have shown that abnormal ventricular wall motion is associated with poor prognosis (cardiac death, non-fatal myocardial infarction, heart failure) in cardiovascular disease (22–25). There are also studies showing that wall motion is associated with poor prognosis in patients with DCM, but most of them merely analyzed the wall motion of each segment obtained by echocardiography, radionuclide imaging and left ventriculography (26–28). However, at present, no study has used SMS to evaluate the prognosis of DCM. Gated SPECT imaging has unique capabilities to provide accurate, reproducible and operator independent quantitative data (29). This study evaluated the prognostic value of the SMS obtained by gated SPECT MPI in patients with DCM. After taking SMS-LVEF interaction into account and correcting for potential confounders (age, gender, BMI, hypertension, diabetes, hyperlipidemia, QRS duration, NYHA class, ACE inhibitors/ARBs, *β*-blockers, diuretics, digoxin, LVEF measured by echocardiography, EDV, ESV, LVEF, STS, PBW, Mean, PSD and PE), we still confirmed that SMS was an independent predictor of cardiac death in patients with DCM. Previous studies (18, 30, 31) found that LV dyssynchrony parameters are predictors of cardiac death in DCM patients. However, the present study suggested that these LV dyssynchrony parameters (such as PE shown in Table 4) are likely not independent predictors of cardiac death in DCM patients. Other factors, such as patient selection and study methods, may also lead to disagreement in different studies. These issues warrant further studies. In addition, we found that age and *β*-blockers were also influential factors for cardiac death in DCM, which is consistent with previous findings (32, 33).

The pathophysiological mechanisms of segmental ventricular wall motion abnormalities in DCM are unclear. Based on previous studies, we considered that several factors may be involved in the mechanisms. First, LV structural and functional parameters, including partial volume effects caused by myocardial wall thinning and increased local wall stress, reduction of blood perfusion due to cardiomyocyte loss, increase of intra-diastolic myocardial pressure, and myocardial fibrotic changes (26, 34, 35), may be mechanisms of abnormal segmental ventricular wall motion. Second, regional myocardial sympathetic denervation and/or hyperinnervation may partially contribute to the abnormalities of segmental ventricular wall motion, because spatiotemporal changes of cardiac sympathetic innervation often occur in cardiac diseases including DCM and these neuronal changes would inevitably affect myocardial contractility, wall stress and motion, and tension of coronary arteries. The severity of these changes is partially associated with local ventricular wall motion abnormalities and myocardial perfusion abnormalities (36). Third, microcirculatory dysfunction may also be contributable to segmental ventricular wall motion. Coronary microvascular damage, loss of endothelium-dependent relaxation of coronary microvessels and inflammation due to viral cardiomyopathy have been associated with segmental ventricular wall motion abnormalities (37–39).

Our study also found the incremental value of SMS in predicting cardiac death in DCM. Compared to a model with only clinical variables (age, gender, BMI, hypertension, diabetes, hyperlipidemia, QRS duration and NYHA class), adding SMS increased the global Chi-square from 12.845 to 28.120 (*P *< 0.001) (shown in Figure 4); adding conventional LV function parameters (EDV, ESV, LVEF, STS, mean and PE) into the model increased the global Chi-square from 12.845 to 42.624 (*P* < 0.001) (Figure 4). The addition of SMS continued to improve the global Chi-square compared to a model that included both clinical variables and conventional LV function parameters (42.624 vs. 44.240, *P* = 0.047) (Figure 4). We also found that the higher the SMS, the worse the prognosis of the DCM patients. These findings suggest that for patients with DCM, in addition to the conventional clinical and imaging indicators, additional analysis of the LV function parameters, especially the SMS, may help clinicians to better assess the conditions of patients and may provide better guidance for patient treatment, and thus may prolong the survival of patients.

In the follow-up of the 81 DCM patients for 44 (25, 54) months, we found that the AUC of the ROC curve of SMS was relatively stable around 0.80 at all timepoints, suggesting that SMS is relatively stable in predicting cardiac death. It is well known that the LV global functional parameter LVEF is an important predictor of cardiac death in patients with DCM (2, 17). The global and regional functions are closely related and the correlation between global systolic wall motion score measured by echocardiography (echocardiographic wall motion score index, WSMI) and LVEF obtained by radionuclide ventriculography is high (*r* = 0.72) (40, 41). Our present study showed a higher correlation between LVEF and SMS (*r* = 0.917), possibly because both of the two parameters were obtained through gated SPECT MPI and were consistent. Our study also showed a reduction in LVEF in the HSMS group compared to the LSMS group (21.5% vs. 12.0%, *P *< 0.001). However, LVEF reflects the global function, and the hypercontractile cardiomyopathy segments may offset the hypokinetic cardiomyopathy segments, thereby preserving total LV systolic function (42). Studies have reported that WSMI is a stronger powerful predictor of adverse cardiac outcomes in patients with acute myocardial infarction (43–45). In addition, WSMI is the only significant independent predictor of cardiac events in patients with chronic congestive heart failure (41). The present study also showed similar results. For patients with DCM, SMS and LVEF were both predictors in the univariate model, while only SMS was an independent predictor of cardiac death in the multivariate model. Here we also found that there was no significant difference in LVEF_echo_ between the survivor group and the cardiac death group, while there was a significant difference in LVEF between the two groups as measured by gated SPECT MPI. The reason for this phenomenon may be that echocardiography is more affected by the operator, and gated SPECT MPI is less affected by the operator and show higher reliability.

Interestingly, we found that the AUC of the ROC curve for SMS was larger than that of LVEF at the 12th month after follow-up, suggesting that SMS is more predictive of early cardiac death in DCM. One possible explanation of this phenomenon is that compensatory hyperkinesia of non-involved myocardium may not affect the ventricular wall motion score while limit the overall LVEF reduction, therefore the sensitivity of the ventricular wall motion score is higher than LVEF in detecting myocardial injury, especially in the early stage of cardiac diseases (46, 47).

## 5. Limitations

Our study had some limitations. First, the study was retrospective with recall bias. Second, the study was a single-center study, thus the findings might not be generalizable to a broad population. Third, the relatively smaller sample size may have limited the statistical power. Forth, this study used a relatively high tracer dose and an all-purpose gamma camera. In the future, we may improve these limitations by conducting similar studies in multicentered and larger population using new equipment.

## 6. Conclusion

We report for the first time that SMS can independently predict cardiac death in patients with DCM and provide incremental prognostic value. These findings may be clinically important to anticipate patients’ conditions in advance, to increase clinical attention, and to prolong patient survival. In addition, we found that SMS might have higher predictive value than LVEF for early cardiac death.

## Data Availability

The original contributions presented in the study are included in the article/Supplementary Material, further inquiries can be directed to the corresponding author/s.
